# Subcutaneous Immunization with Unaltered Axenic Malaria Parasite Liver Stages Induces Sterile Protection against Infectious Sporozoite Challenge

**DOI:** 10.3390/vaccines10111884

**Published:** 2022-11-08

**Authors:** Mohd Kamil, Gozde Deveci, Umit Y. Kina, Stefan H. I. Kappe, Ahmed S. I. Aly

**Affiliations:** 1 Aly Lab., Beykoz Institute of Life Sciences and Biotechnology, Bezmialem Vakif University, Istanbul 34820, Turkey; 2 Department of Biotechnology, Institute of Health Sciences, Bezmialem Vakif University, Istanbul 34093, Turkey; 3 Center for Global Infectious Disease Research, Seattle Children’s Research Institute, 307 Westlake Avenue N. Suite 500, Seattle, WA 98109, USA; 4 School of Science and Engineering, Al Akhawayn University, Ifrane 53000, Morocco

**Keywords:** malaria, vaccine, axenic liver stage, subcutaneous immunization, *Plasmodium yoelii*

## Abstract

Host cell-free, axenic development of liver stages (LS) of the malaria parasite has been demonstrated. Here we explored axenic liver stages as a novel live whole parasite malaria vaccine platform, which is unaltered and not prone to human-error, compared to the immunization with live-attenuated sporozoites that must be done intravenously. We show that in contrast to live sporozoites, axenic LS are not infectious to the immunized host. Subcutaneous immunizations of mice with *Plasmodium yoelii* axenic LS, developed from wild-type (WT) sporozoites or WT sporozoites expressing enhanced-GFP, conferred sterile protection against *P. yoelii* infectious sporozoite challenge. Thus, axenic liver stages of *P. falciparum* and *P. vivax* might constitute an attractive alternative to live sporozoite immunization.

## 1. Introduction

While recent intervention strategies have led to the reduction in malaria burden, malaria remains a major cause of morbidity and mortality, especially in children under 5 years of age living in endemic areas. The global death estimate by malaria is about 405,000 peoples annually [1]. The World Health Organization (WHO) has reported that the rate of decline in malaria cases has slowed to 2% in the years 2015 to 2019, in comparison to 27% decline between 2000 to 2015 [2]. The predominant cause of this increase in malaria cases is the generation of drug resistance [3]. Therefore, an effective vaccine would be an important addition to the arsenal required to further reduce the number of malaria related deaths. 

The malaria parasite pre-erythrocytic stage, which includes the mosquito-inoculated sporozoites and the liver stages (LS), is a clinically-asymptomatic life cycle stage of the malaria parasite, that precedes the pathogenic blood stage, which causes the malaria disease [4,5,6,7,8]. Therefore, the pre-erythrocyte stage is considered as a prime target for anti-infection malaria vaccine development [8,9,10]. Noteworthy, till now only live attenuated pre-erythrocytic stage vaccines have shown to provide sterilizing immunity against malaria [11,12,13]. Attenuation of live pre-erythrocytic stages has been achieved so far by gamma- or X-rays irradiation, by genetic deletion of LS essential genes. These methods can be prone to human errors in application, design or transfer, and are largely limited in their protective immunity to the attenuated strain used. Moreover, the injection of sporozoites that must be live and capable of invasion is a tremendous labor- and resource- intensive effort, given that they must be purified out of mosquitoes reared under aseptic conditions, and transferred in a frozen state. Importantly, the route of vaccination that is mandatory for this approach to show promise of efficacy is the intravenous route, which is not a practical route for mass-use in humans living in endemic areas. 

In this study, we tried to address some of the above-mentioned challenges for whole-parasite live malaria vaccines by the generation of in-vitro developed LS from WT (wild-type) or WT-like *Plasmodium yoelii* sporozoites [14], and we used them as a foolproof and safe, live whole-parasite vaccine platform in subcutaneous immunizations that conferred sterile protection against infectious salivary gland sporozoites challenge. 

## 2. Materials and Methods

### 2.1. Animals and Parasite

6–8-week-old female BALB/c mice were purchased from the animal service facility of Bezmialem Vakif University. The non-lethal *P. yoelii* 17X-NL WT and *P. yoelii* 17X-NL *p230p(−)* strains were stored as frozen stock at −150 °C. *Pyp230p(−)* is a eGFP (enhanced green fluorescent protein) expressing WT-like parasite strain, which was genetically modified to express eGFP in all life cycle stages, under the control of *PyHSP70*_1 constitutive promoter, as a knockout replacement of the dispensable gene *Pyp230p*. Targeted deletion of P230p did not show any phenotypic effect as described previously [15]. Freshly thawed parasites were injected into donor naive mice for mosquito feeding to get sporozoites as described earlier [16]. 4–8-day old female *Anopheles stephensi* mosquitos were allowed to blood-feed on anesthetized female BALB/c mice infected with both strains of parasites. Blood fed mosquito were maintained at 24 °C, 75% humidity. At day 15 post infection, mosquitoes were collected, rinsed with 70% ethanol for 10 s, and washed twice in sterile medium to reduce contamination before dissection. 

### 2.2. Salivary Gland Sporozoite Collection and In-Vitro Transformation

On day 15 post infection, salivary glands were dissected in sterile culture media containing DMEM (Gibco, cat#11965092) supplemented with 10% FBS (Gibco, cat#10082147), 500U/mL penicillin–streptomycin (Gibco, cat#15070063), and 1.50 µL/mL fungizone (Gibco, cat#15290018). The dissected glands were ground using a pestle and spun at 800 rpm for 3 min. Supernatant containing sporozoites were collected, and the number of sporozoites were determined using hemocytometer. For transformation 200,000 sporozoites per well in a 24-well culture plates were dispersed in 500 µL complete medium. The sporozoites were incubated at 37 °C temperature in 5% CO_2_ in a humidified incubator. The transformation of sporozoites into early liver stage were observed by live fluorescence microscopy at different time intervals using eGFP expressing WT-like *P. yoelii*. 

### 2.3. Live Fluorescence Imaging

The conversion of salivary gland sporozoites into early liver stage was confirmed by live fluorescence imaging of cultured eGFP expressing sporozoites. Transformed parasites were harvested at 6 hrs (6 h), 12 hrs, 18 hrs and 24 hrs time intervals and images were captured using confocal laser scanning microscope Leica TCS SP8.

### 2.4. Infectivity of Sporozoites and Axenic LS in mice

6 to 8-week-old female BALB/c mice were used to check the infectivity of axenically developed early LS. Axenic LS from 500,000 and 1,000,000 cultured day-14 salivary gland sporozoites doses, and day 15 freshly-dissected salivary gland sporozoites doses were injected into groups of 3 BALB/c mice per group. After 24 hrs of incubation, transformed parasites were harvested, and collected by centrifugation at 7000 rpm for 1 min. The doses were prepared in 200 µL with incomplete sterile RPMI media for axenic LS and sporozoite doses (Gibco, cat#a1049101), and injected intravenously into mice. Blood stage infection was monitored from day 3 to day 15 post sporozoite injection by Giemsa-staining of thin blood smears from tail vein. 

### 2.5. Vaccination

6–8-week-old female BALB/c mice were used to check the efficacy of axenically developed early LS vaccine candidate. 200,000 cultured sporozoites were used as a single axenic LS vaccine dose. After 24 hrs of incubation, transformed parasites were harvested, and collected by centrifugation at 7000 rpm for 1 min. The supernatant was removed and remaining 200 µL was resuspended to vaccinate mice. Subcutaneous (SC) (n = 10 mice per group for both *Py*WT and *Pyp230p(−)*) route was used to immunize mice. Four immunizations were performed at a three-week interval between prime (0 day), first boost (21 days), second boost (42 days) and third boost (63 day). 

### 2.6. Parasite Challenge

The efficacy of immunization was checked by challenge with day 14/15 *P. yoelii* infectious salivary gland sporozoites. Mice were intravenously injected with *P. yoelii* 17X-NL salivary gland sporozoites resuspended in incomplete RPMI media. Sporozoites were prepared as described earlier [16]. Eight weeks after the final immunization, each mouse was injected via the tail vein with 200 µL of RPMI media containing 500 salivary gland sporozoites. Two groups of five naive mice each were also used as control groups in parasite challenge study. After parasite challenge, blood stage infection was monitored from day 3 to day 15 by Giemsa staining of tail vein thin blood smears. Protection was defined as the complete absence of erythrocytic stage parasitemia on day 15 post challenge

## 3. Results

### 3.1. In Vitro Cultured Sporozoites Transform into Early Liver Stages within 24 hrs of Incubation

**Figure 1:**
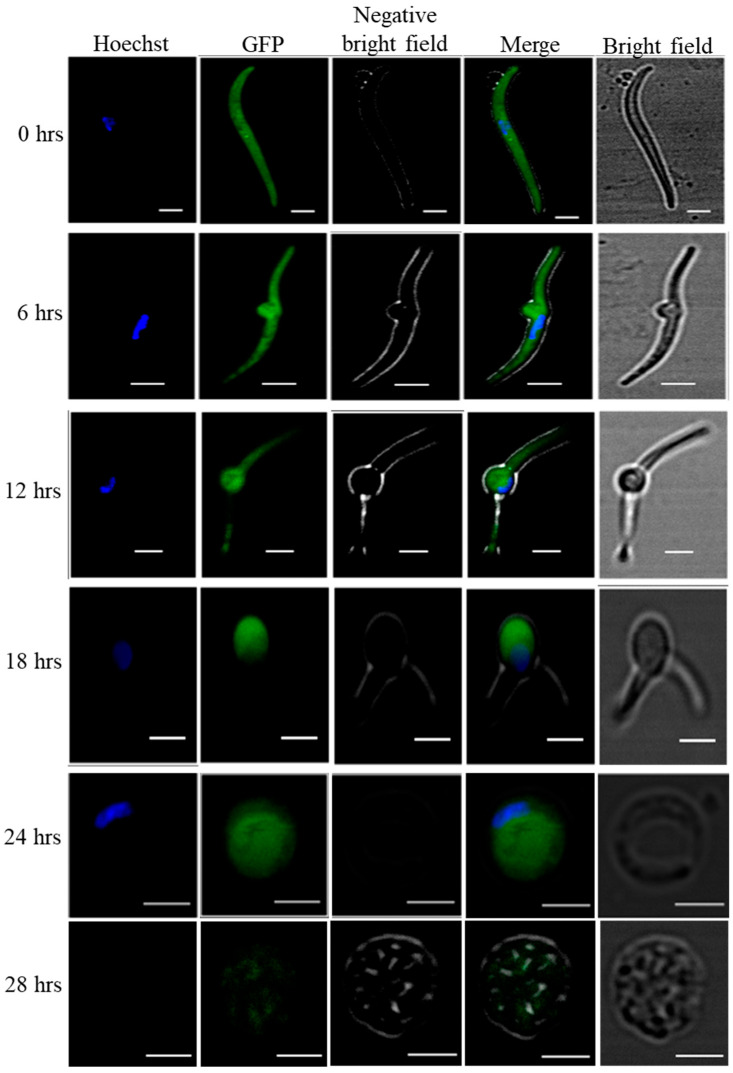
Axenic transformation of eGFP expressing *P. yoelii p230p(−)* salivary gland sporozoites into LS. In-vitro host cell free cultured salivary gland sporozoites completely transform into early LS after 24 hrs of incubation. To follow the cell free transformation of salivary gland sporozoites into early exoerythrocytic forms, we used eGFP expressing *Pyp230p(−)* and followed the transformation by confocal microscopy of cultured sporozoites at 6 hrs intervals for 24 hrs. At 28th of Axenic LS culture we could not find any good GFP images. Scale bars 2 µm.

To check the transformation of salivary gland sporozoites into early exoerythrocytic forms, we used WT-like eGFP expressing *P. yoelii* strain *Pyp230p(−)* [15]. The live fluorescence microscopy clearly shows that the transformation started within the 6 hrs of incubation where cultured sporozoites developed into transformation bulbs, and at 24 hrs of incubation the cultured sporozoites completely developed into early LS. (Figure 1). 

### 3.2. Immunization with Cell-Free Developed Early Liver Stages is Safe and Provides Sterile Protection

Safety of the vaccine is a major concern when developing attenuated whole organism malaria vaccine [17]. Therefore, the axenic LS transformation was followed by the infectivity test of in-vitro cell free developed early LS. 500,000 and 1,000,000 sporozoites were cultured per well and were harvested 24 hrs later and intravenously injected into 6-7 weeks old BALB/c mice. The infectivity was followed by Giemsa staining of tailed blood smearing from day 3 to day 15 post infection. We did not observe any blood stage parasite until day 15 post infection, in contrast to mice injected with WT or WT-like sporozoites that developed all parasitemia by day 4 post intravenous sporozoite injection. Hence, axenically developed early LS were considered as a safe whole organism vaccine candidate. The scheme of the infectivity test is given in Table 1. 

Subsequently, Immunization was performed using axenic LS which demonstrated strong protection against salivary gland infectious sporozoites infection. Mice were immunized with the harvested axenic LS using subcutaneous route of immunization (n = 10 mice per group for both *P. yoelii* WT and *P. yoelii p230p(−)*). The vaccinated mice were intravenously challenged with 500 *P. yoelii* 17X-NL salivary gland sporozoites eight weeks after the final immunization to check the effectiveness of vaccine candidate. A 100% sterile protection was confirmed with no blood stage infection observed until day 15 post sporozoite challenge. The immunization scheme is summarized in Table 1.

## 4. Discussion

Immunization using whole attenuated sporozoites is the only vaccination strategy which provides complete long-lasting protection in human subjects [18]. Different approaches have been applied to develop whole parasite vaccine (WPV) using sporozoites, including radiation [19], genetically-attenuated [20] and infectious sporozoites concurrent with antimalarial drug treatment cover [21,22]. However, all these methods have their own limitations and are prone to human error in attenuation method, attenuation design, transfer of attenuated parasites or application of drug cover, and are therefore not entirely foolproof [23]. Importantly, it has been shown so far that the protective immunity developed following immunization with whole parasite vaccine are largely strain specific. Since these attenuation methods are extremely labor- and resource-intensive and need to be applied intravenously in order to be effective, therefore it is not foreseeable that these attenuation methods can be applied systematically to target geographically-distinct malaria parasite strains.

Therefore, herein we report a more practical and foolproof method of attenuation that does not involve alteration of the parasite or treatment of the host. The axenically-developed LS vaccine candidate is completely safe and highly efficacious with a widely accepted subcutaneous (SC) route of immunization and shows sterile protection against infectious sporozoite challenge in the immunized mice. Though, studies show WSV elicit primarily CD8^+^ T cell mediated protection, [24,25,26], it will be an important goal for future studies to identify the type of protective immunity induced by axenically developed LS as a WPV, as the subcutaneous inoculation might possibly induce humoral immunity.

## 5. Conclusions

Herein, we are showing that axenically developed early LS parasites are safe and provide sterile protection against salivary gland sporozoites challenge in immunized mice. Our data also supports future work toward testing our malaria vaccine in other animal models of malaria infection such as nonhuman primate models. This future work will be critical to support future human clinical trials using vaccines targeting clinically asymptomatic stage and achieving the World Health Organization’s goal of malaria eradication by 2030. 

## Figures and Tables

**Table 1:** Infectivity and Immunizations with axenically developed *P. yoelii* LS.

**Infectivity of Axenic LS vs. Salivary Gland Sporozoites in BALB/c Mice**				**Sterile Protection against Malaria Parasite Sporozoite Challenge Following Subcutaneous Immunization with Axenic LS**				
**Group (n)**	**Infection Dose**	**Route**	**Infected/** **Injected**	**Group (n)**	**Immunization Dose**	**Immunization Intervals**	**Route**	**Protected/** **Challenged**
**Group A: *Pyp230p(−)*** **Axenic LS** **(3)**	500,000	IV	0/3	**Group 1:** ***Pyp230p(−)*** **Axenic LS** **(10)**	200,000	0, 21, 42, 63	SC	10/10
**Group B: *Pyp230p(*** *−**)*** **Axenic LS** **(3)**	1,000,000	IV	0/3	**Group 2: PyWT** **Axenic LS** **(10)**	200,000	0, 21, 42, 63	SC	10/10
**Group C: *Pyp230p(*** *−**)*** **Sporozoites** **(3)**	500	IV	3/3	**Group 3: Uninfected** ***Anopheles stephensi*** **salivary glands (5)**	N/A	0, 21, 42, 63	SC	0/5
**Group D: PyWT** **Axenic LS** **(3)**	500,000	IV	0/3	**Group 4: Naive Control** **(5)**	N/A	N/A	N/A	0/5
**Group E: PyWT** **Axenic LS** **(3)**	1,000,000	IV	0/3	N/A	N/A	N/A	N/A	N/A
**Group F: PyWT** **Sporozoites** **(3)**	500	IV	3/3	N/A	N/A	N/A	N/A	N/A

## Data Availability

Not applicable.
